# Aerodynamic Optimization of Relay Nozzle Using a Chebyshev KAN Surrogate Model Integration and an Improved Multi-Objective Red-Billed Blue Magpie Optimizer

**DOI:** 10.3390/biomimetics11040282

**Published:** 2026-04-18

**Authors:** Min Shen, Ziqing Zhang, Guanxing Qin, Dahongnian Zhou, Lizhen Du, Lianqing Yu

**Affiliations:** 1 Three Dimensional Textile Hubei Engineering Research Center, Wuhan Textile University, Wuhan 430200, China; 2 School of Mechanical Engineering and Automation, Wuhan Textile University, Wuhan 430200, China

**Keywords:** relay nozzle, aerodynamic optimization, surrogate model, improved Multi-objective Red-billed Blue Magpie Optimizer (IMORBMO), Chebyshev polynomial Kolmogorov–Arnold Network (Chebyshev KAN)

## Abstract

In air jet looms, relay nozzles are critical components in governing airflow velocity and air consumption during the weft insertion process. Although computational fluid dynamics (CFD) offers high-fidelity simulation for aerodynamic analysis, its computational burden hinders its practicality in iterative aerodynamic design of relay nozzles. To address the challenge, this study proposes a data-driven framework integrating a Chebyshev polynomial Kolmogorov–Arnold Network (Chebyshev KAN) surrogate model with an Improved Multi-objective Red-billed Blue Magpie Optimizer (IMORBMO). The accuracy of the Chebyshev KAN model was benchmarked against conventional multilayer perceptrons (MLP), convolutional neural networks (CNN), and the standard Kolmogorov–Arnold Network (KAN). Experimental results demonstrate that the Chebyshev KAN model achieves the lowest mean absolute error (MAE) of 0.103 for airflow velocity and 0.115 for air consumption. Building upon the non-dominated sorting and crowding distance strategies, IMORBMO was developed, incorporating an adaptive mutation mechanism by information entropy for improvement of convergence, diversity, and uniformity of the Pareto-optimal solutions. Comprehensive evaluations on the ZDT and WFG benchmark suites confirm that the IMORBMO consistently attains the best and highly competitive performance, yielding the lowest generation distance (GD), inverted generational distance (IGD) values and the highest hypervolume (HV). Applied to the aerodynamic optimization of a relay nozzle, the proposed framework delivers an optimal aerodynamic design that increases airflow velocity by 10.5% while reducing air consumption by 15.4%, as verified by CFD simulation. The steady-state flow field was simulated by solving the Reynolds-Average NavierStokes equations with the *k*–*ω* turbulent model, utilizing Fluent 2025.R2. No-slip wall, inlet pressure and outlet pressures are boundary conditions to the relay nozzle surfaces. This work establishes a computationally efficient and accurate optimization paradigm that holds significant promise for aerodynamic design and other complex real-world engineering applications.

## 1. Introduction

Air jet looms are the most prevalent type of weaving machinery, favored for their high productivity and precise control. However, their substantial energy consumption significantly increases operational costs for textile manufacturers [1]. In air jet looms, there are a main and multiple relay nozzles that collectively generate a high-velocity airflow to propel a weft yarn across the shed. Among these components, the relay nozzle plays a critical role, as it directly influences both the airflow velocity and air consumption [2]. In response to the dual pressures of global economic expansion and the reduction of carbon dioxide emissions, it is essential to aerodynamically optimize the design of relay nozzles for enhancing insertion efficiency and saving energy. However, there is a lack of innovative optimization frameworks that can achieve substantial air consumption reduction without compromising the airflow velocity of the relay nozzle.

Computational fluid dynamics (CFD) offers a powerful tool for investigating the airflow distribution of a relay nozzle. Existing approaches rely heavily on the airflow characteristics of the relay nozzle. Adámek [3] optimized the exit shape of the relay nozzle to enhance airflow speed. Belforte et al. [4] investigated the effect of outlet area on the airflow velocity of the relay nozzle. Delcour et al. [5] examined the effect of exit shape on transient characteristics of airflow. More recently, Xiao et al. [6] explored the influence of outlet geometry on the intersecting airflow velocity in the profiled channel. However, CFD simulation requires high-performance computer resources due to the high-dimensional, nonlinear nature of turbulent flow. This substantial computational cost limits the practicality of the CFD technique in iterative aerodynamic optimization of relay nozzles [7].

To alleviate computational costs and accelerate design cycles, a surrogate model-based aerodynamic optimization method has emerged as a promising alternative [8]. A surrogate model is a data-driven approximation that replaces computationally expensive numerical simulation by leveraging mathematical expressions, polynomial fitting, regression models, or neural networks [9]. Due to advances in machine learning (ML) and deep learning (DL), researchers have focused on developing various DL-based surrogate models that achieve both high precision and efficiency in aerodynamic design [10]. Representative surrogate models include artificial neural network (ANN) [11], convolutional neural networks (CNN) [12], deconvolutional neural network (DeCNN) [13], and variational autoencoders (VAEs) [14]. However, these traditional multilayer perceptrons (MLP) surrogate models typically rely on a set of predefined activation functions prior to training the neural network and apply uniformly across all neurons [15]. This static choice mechanism leads to limitations such as neuron saturation and vanishing gradients in high-dimensional regression analysis.

To address this limitation, Liu et al. [16] firstly developed an innovative Kolmogorov–Arnold Networks (KAN) as an alternative to traditional multilayer perceptrons (MLPs). Unlike MLPs, KAN architecture places learnable activation functions on the edges rather than within the neurons, enabling nonlinear transformation to be performed directly through these edge-based activation functions [17]. This mechanism of KAN can mitigate the curse of dimensionality in high-dimensional data, making KAN well-suited for complex nonlinear regression tasks. However, KAN is prone to overfitting in high-dimensional nonlinear regression problems [18].

Aerodynamic optimization design inherently involves multiple conflicting objectives, along with a large number of design variables. In such scenarios, traditional single-objective optimization algorithms are inadequate for dealing with the trade-off in practical aerodynamic design [19]. Consequently, there is a growing demand for robust multi-objective (MO) algorithms capable of identifying a set of solutions commonly referred to as the Pareto front (PF). For solving aerodynamic design problems, some researchers have developed a variety of metaheuristic MO algorithms, such as the non-dominated sorting genetic algorithm (NSGA) and its enhanced variant NSGA-II [20], multi-objective evolution (MOEAD) [21], multi-objective whale optimization [22], multi-objective dung beetle optimization algorithm (MODBO) [23], and multi-objective dragonfly (MODA) [24]. More recently, Ouyang et al. [25] introduced a Multi-objective Red-billed Blue Magpie Optimizer (MORBMO) inspired by the hunting behavior and food storage behaviors of red-billed blue magpies. However, existing MO algorithms still face significant challenges in maintaining solution diversity across the Pareto front in high-dimensional design space. This issue is called “curse of dimensionality”, which not only degrades the search efficiency but also dramatically increases computational costs. Hence, the MO optimization algorithms tend to suboptimal or sparsely distributed solutions, limiting their applicability to engineering aerodynamic design tasks.

Notably, research on the multi-objective optimization algorithms integration with surrogate models for high-dimensional nonlinear aerodynamic design remains scarce in the current literature. This gap underscores the need for developing a robust multi-objective optimization algorithm integration with a high-accuracy surrogate model, effectively balancing convergence, diversity, and efficiency in complex aerodynamic design. This study aims to propose a novel data-driven optimization framework that integrates an Improved Multi-objective Red-billed Blue Magpie Optimizer (IMORBMO) based on a Chebyshev Kolmogorov–Arnold Network (Chebyshev KAN) surrogate model for aerodynamic design of a relay nozzle. The proposed framework has the potential to substantially improve prediction accuracy and convergence compared to conventional approaches.

The main contributions are summarized as follows:

(1) A Chebyshev KAN surrogate model is constructed by replacing B-spline basis functions with Chebyshev polynomials as learnable activation functions, enabling highly accurate approximation of the nonlinear mapping between high-dimensional structural design parameters and the nonlinear aerodynamic response of the relay nozzle.
(2) The effectiveness of the Chebyshev KAN model is evaluated against established surrogate models, including multilayer perceptron (MLP), convolutional neural networks (CNN), and the original B-spline based Kolmogorov–Arnold Network (KAN).
(3) A data-driven framework integrates a Chebyshev KAN surrogate model with an Improved Multi-objective Red-billed Blue Magpie Optimizer (IMORBMO), which incorporates an adaptive mutation mechanism based on information entropy for improved convergence and solution diversity in high-dimensional aerodynamic design problems.
(4) Comparative experiments have been conducted to validate the effectiveness of the proposed IMORBMO with six state-of-the-art MO on standard benchmark functions. Moreover, the optimal aerodynamic design geometric parameters of the relay nozzle were obtained using the proposed IMOBMBO algorithms based on the Chebyshev KAN surrogate model.

The remainder of this article is organized as follows: Section 2 introduces the Chebyshev KAN surrogate model and its formulation; Section 3 presents the general framework of an Improved Multi-objective Red-billed Blue Magpie Optimizer (IMORBMO); Section 4 outlines comparative experiments assessing the predictive accuracy of CNN, MLP, KAN and the proposed Chebyshev KAN surrogate model; in Section 5, the integrated Chebyshev KAN and IMORBMO framework is applied to optimize the aerodynamic performance of the relay nozzle, followed by an in-depth discussion; finally, Section 6 summarizes key insights drawn from the Pareto-optimal solutions for the relay nozzle in the air jet loom.

## 2. Surrogate Model for Prediction of Aerodynamic Performance

### 2.1. Chebyshev Polynomial Kolmogorov–Arnold Networks

To optimize the aerodynamic geometry of the relay nozzle, there is a need to establish an accurate surrogate model for predicting both the nonlinear airflow velocity and air consumption instead of using traditional CFD simulation. Recently, the Kolmogorov–Arnold Networks (KAN) was proposed, which uses B-spline basis functions in the initial formulation. However, B-splines possess localized support, being non-zero only within a finite interval. This locality hinders KAN from capturing the relationships between high-dimensional structural features and the nonlinear aerodynamic response of the relay nozzle. As a result, the approximation error tends to grow exponentially as dimensionality increases.

To address these limitations, we propose a novel variant termed Chebyshev Kolmogorov–Arnold networks (Chebyshev KAN) by replacing the B-spline basis function with the Chebyshev polynomial. This is due to desirable properties of Chebyshev polynomials, such as fast convergence and minimizing the Runge phenomenon, which is the large oscillations observed near the edges of the interval using high-degree polynomials. The fundamental cause of the Runge phenomenon is that when using equidistant nodes for high-degree polynomial interpolation, the error increases sharply near the endpoints of the interval, leading to severe oscillations. The roots of Chebyshev polynomials, termed Chebyshev nodes, exhibit a non-uniform distribution over the interval [−1, 1], characterized by a higher density near the endpoints and sparsity in the center. This specific arrangement effectively counteracts the error amplification typically observed near the boundaries, thereby suppressing the oscillatory behavior of the interpolating polynomial and guaranteeing stability and convergence, even in high-degree interpolation. This property is well-suited for high-dimensional, nonlinear regression tasks due to its global support and spectral convergence properties.

Both B-spline KAN and Chebyshev KAN are grounded in the Kolmogorov–Arnold representation theorem that any continuous multivariate function ${f:[0,1]}^{n}\to R$ can be represented as a superposition of continuous univariate functions. The theorem guarantees the existence of a decomposition as a mathematical formulation:

(1) $$f(x)=\sum_{q=1}^{2n+1}\Phi_{q}(\sum_{q=1}^{n}\psi_{p,q}(x_{p}))$$

where $\psi_{p,q}\;$ are one-dimensional univariate functions, and $\Phi_{q}$ composes these functions to reconstitute f(x).

In practice, this study adopts Chebyshev polynomials as the basis function $\psi_{p,q}$ to enhance express ability for nonlinear input features, such as:

(2) $$\psi_{p,q}(x)=\sum_{n=1}^{N}\left(w_{p,q}\xi (x)+{\tilde{w}}_{p,q}\Gamma_{p,q}(x)\right)+\beta (x)$$

where $\Gamma_{p,q}(x)$ is a learnable activation function, ${\tilde{w}}_{p,q}$ is a learnable parameter to adjust the contribution of the term $\Gamma_{p,q}(x)$, and β(x) is a bias term.

Let  Γ(x) be an analytic representation, and its Chebyshev polynomial of the first kind be defined as:

(3) $$\Gamma_{p,q}\left(x\right)=cos(n.\;arccos(2x-1))$$

where n is the degree of the polynomial, $x\in \left[-1,1\right]$.

The coefficient of the Chebyshev polynomial is replaced with a learnable parameter $w_{p,q,n}$, allowing the network to adaptively shape the activation function during training. Consequently, the nonlinear activation function can be expressed in a simplified form as:

(4) $$\psi_{p,q}\left(x\right)=\sum_{n=1}^{N}{w_{p,q,n}\left(x\right)}^{n}+\beta_{q}$$

where $\beta_{q}$ is the bias coming from the Chebyshev series.

**Figure 1:**
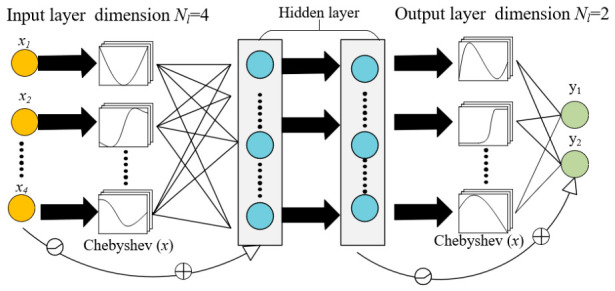
Schematic of the Chebyshev KAN surrogate model.

The diagram of the Chebyshev KAN surrogate model is illustrated in Figure 1. The Chebyshev KAN can provide dual guarantees of prediction accuracy and robustness, thereby enhancing the reliability and practicality of aerodynamic performance optimization.

### 2.2. Evaluation Metrics for Chebyshev KAN Surrogate Model

To verify the prediction accuracy of the Chebyshev KAN surrogate model, three different evaluation indicators were adopted, that includes the mean absolute error (*MAE*), root mean square error (*RMSE*), and coefficient of determination (*R*^2^). These calculation formulas are as follows:

(5) $$MAE=\frac{1}{N}\sum_{i=1}^{N}\left|y_{i}-{\hat{y}}_{i}\right|$$

(6) $$RMSE=\sqrt{\frac{1}{N}\sum_{i=1}^{N}(y_{i}-{\hat{y}}_{i}{)}^{2}}$$

(7) $$R^{2}=1-\frac{\sum_{i=1}^{N}(y_{i}-{\hat{y}}_{i}{)}^{2}}{\sum_{i=1}^{N}(y_{i}-\bar{y}{)}^{2}}$$

In the equations above, $y_{i}$ represents the actual value, ${\hat{y}}_{i}$ denotes the predicted value, and *N* signifies the number of samples.

## 3. An Improved Multi-Objective Red-Billed Blue Magpies Optimizer (IMORBMO)

### 3.1. Multi-Objective Problem Definition

**Figure 2:**
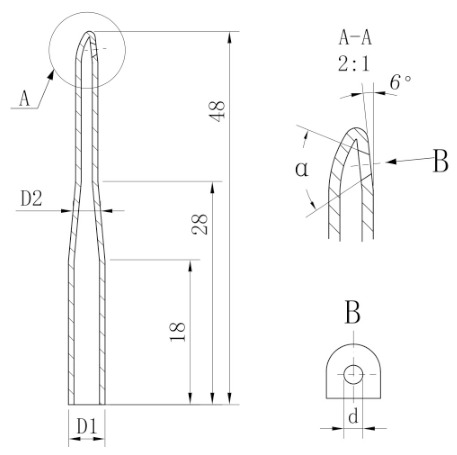
Geometric models of the relay nozzles.

This section describes the processes of the Multi-objective Red-billed Blue Magpie Optimizer (MORBMO). The first objective is to reduce air consumption *Q,* while the secondary objective is to maximize the airflow speed *V* of the relay nozzle. Therefore, the negative of the velocity is taken as the optimization objective to be minimized. The structural characteristics comprise the outlet diameter of the inlet diameter (*D*1 mm), straight pipe diameter (*D*2 mm), diameter of circular exit (*d* mm), and conical degree (rad), as depicted in Figure 2.

The structural parameter of the relay nozzle, namely *D*1, *D*2, *d*, α and output variables *V* and *Q,* constitute a six-dimensional coupled space, ${K\subset R}^{6}$. Incorporate the predicted value of $Q_{i}$ and $V_{i}$ of Chebyshev KAN surrogate model into the MORBMO to construct a robust aerodynamic performance optimization framework. The formulation of the mathematical model is presented below:

(8) $$\left\{\begin{array}{l} f_{1}\left(X\right)=min\left(Q_{i}\right)\;\;\;\;\;\;\;\;\;\;\;\;\;\;\;\;\;\;\;\;\;\;\;\;\;\;\;\;\;\;\;\;\;\;\;\;\;\;\;\;\;\;\;\;\;\;\\ f_{2}\left(X\right)=max\left(\left|V_{i}\right|\right)\;\;\;\forall X=\left\{X_{1},X_{2},\ldots ,X_{m}\right\} \end{array}\right.$$

Subject to:

(9) $$\;\left\{\begin{array}{l} {g_{1}\left(X\right)=d}_{i}^{min}\leq d_{i}\leq d_{i}^{max},\;outlet\;area\;constraints\\ g_{2}\left(X\right)=D_{i}^{min}\leq D_{i}\leq D_{i}^{max},\;inlet\;area\;constraints \end{array}\right.$$

where i=1,2,…m, j=1,2,…n is the number, the superscript max and min indicate upper and lower bounds, respectively.

In contrast to single-objective solutions, which can be straightforwardly assessed using arithmetic comparisons, in MO optimization, there is no “best” solution that satisfies all objectives concurrently. Pareto dominance is employed to evaluate and compare solutions in the search space, where no solution is “dominated” by any other within the set. The concept of “domination” is formally defined through mathematical formulation.

**Definition** **1.** *(Dominance relation) For a minimal problem, p and q are two solutions, respectively. The p dominates q can be defined by:*

(10) $$p≺q\;\;\;\;\;\;\;\;if\left\{\begin{array}{cc} f_{i}\left(p\right)\leq f_{i}\left(q\right), & for\;all\;i=\left\{1,2\right\}\\ f_{j}\left(p\right)<f_{j}\left(q\right), & for\;at\;least\;one\;j=\left\{1,2\right\} \end{array}\right.$$

where i, j is the number of objective functions, $f_{i}\left(p\right)$ and $f_{i}\left(q\right)\;$ is the value of two solutions in the decision vector, respectively.

**Definition** **2.** *(Pareto set). A solution *x *is considered as a Pareto optimal if there exists no other solution *y *that dominates it. The set of all such non-dominated solutions constitutes the Pareto set (PS), which can be expressed in the equation:*

(11) $$PS=\left\{p\in X|∄\;q\in X:p≺q\right\}$$

**Definition** **3.** *(Pareto front). The Pareto front (PF) is the mapping of the Pareto set (PS) into the objective space through the objective functions, defined as:*

(12) $$PF\left\{F\left(X\right)|X\in PS\right\}$$

**Figure 3:**
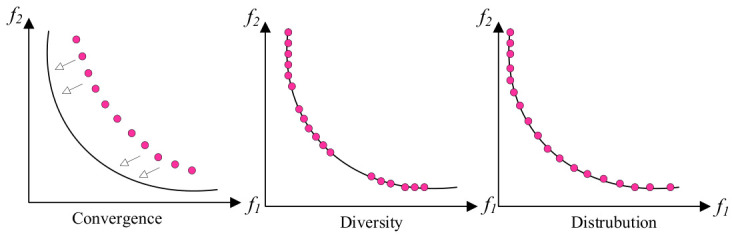
Diagram for convergence, diversity, and distribution performance of the MO algorithm.

The aerodynamic optimization of the relay nozzle problem involves two decision variables that are presented in Figure 3. The red circles represent Pareto-optimal vectors, commonly named the Pareto front, while the black curve represents the true PF. Evaluating MO algorithms involves convergence, diversity, and distribution characteristics, as shown in Figure 3. The primary challenge in MO stems from the inherent conflict among objectives. Consequently, research efforts are typically directed toward the development of specialized MO algorithms capable of generating a well-distributed set of non-dominated solutions that closely approximate the true Pareto front. In this section, we propose an Improved Multi-objective Red-billed Blue Magpie Optimizer for aerodynamic design and optimization of the relay nozzle in an air jet loom.

### 3.2. Red-Billed Blue Magpie Optimizer (RBMO)

The single-objective Red-billed Blue Magpie Optimizer (RBMO) is a bio-inspired metaheuristic optimization algorithm. The RBMO successfully explores and exploits the search space by simulating the behaviors of searching, chasing, attacking prey, and food catching in red-billed blue magpies. The pseudocode for RBMO is provided in Fu et al. [26]. This subsection briefly describes the mathematical model of single-objective RBMO before the deviation of the multi-objective optimizer.

Step 1: Initialization of the population of RBMO

The RBMO starts with the initial population X, and main parameters, including the maximum number of iterations *Max_iter*, the dimensionality of problems, the lower bounds *L,* and upper bounds *U*. An initial population X within the search space is defined in Equation (14).

(13) $$X=\left[\begin{array}{ccc} X_{1,1} & \ldots & X_{1,dim}\\ \vdots & \ddots & \vdots \\ X_{n,1} & \ldots & X_{n,dim} \end{array}\right]$$

where X indicates the location assigned to the search agent, n represents the population size, and dim  indicates the dimensionality of the problem being solved.

A random function generates an initial population within the range *L* to *U*, as described in Equation (14).

(14) $$X^{i}\left(t\right)=\left(U-L\right)\times {Rand}_{1}+L$$

where Rand denotes a randomly generated number between 0 and 1, the lower bounds *L,* and upper bounds *U* of the optimization parameter.

Step 2: Searching for food

While searching for food, red-billed blue magpies typically forage in small groups (2–5 individuals) or large groups of more than 10 individuals to ensure search efficiency. To access food sources, red-billed magpies adopt diverse strategies, including jumping, walking, and terrestrial movement. Small groups of red-billed magpies search for food. Equation (15) is defined as

(15) $$X^{i}\left(t+1\right)=X^{i}\left(t\right)+\left(\frac{1}{p}\sum_{m=1}^{p}X^{m}\left(t\right)-X^{rs}\left(t\right)\right){rand}_{2}\;$$

where *t* is the current number of iterations, $X^{i}\left(t+1\right)$ is the *i*th new search agent position, p∈[2,5] represents the number of red-billed blue magpies hunting in small groups, $X^{m}$ is the *m*th individual chosen at random, $X^{i}$ is the *i*th individual, and $X^{s}\left(t\right)$ is randomly selected search agents in the current iteration.

Group foraging for food in large numbers is given by

(16) $$X^{i}\left(t+1\right)=X^{i}\left(t\right)+\left(\frac{1}{q}\sum_{m=1}^{p}X^{m}\left(t\right)-X^{rs}\left(t\right)\right){rand}_{3}$$

where q∈[10,N] is the search agents in larger number, and it is also randomly selected from the entire population.

Step 3: Attacking prey

Red-billed blue magpies demonstrate hunting expertise and cooperative behavior for pursuing prey. When their primary targets are typically small prey or plants, the mathematical model is defined as Equation (17). They collectively target larger prey such as large insects or small vertebrates. The expression is given in Equation (18)

(17) $$X^{i}\left(t+1\right)=X^{i}\left(t\right)+CF\times \left(\frac{1}{p}\sum_{m=1}^{p}X^{m}\left(t\right)-X^{i}\left(t\right)\right){rand}_{1}$$

(18) $$X^{i}\left(t+1\right)=X^{\beta }\left(t\right)+CF\times \left(\frac{1}{q}\sum_{m=1}^{p}X^{m}\left(t\right)-X^{i}\left(t\right)\right){rand}_{2}$$

where $CF={\left(1-\;t/T\right)}^{2t/T}$, $X^{i}\left(t\right)$ is the position of the food, ${rand}_{1}$ and ${rand}_{2}\in [0,1]$ represents random numbers following a standard normal distribution.

Step 4: Stored food

In addition to searching for and attacking food, red-billed blue magpies also chase excess food in tree hollows or other concealed locations for future consumption, ensuring a stable food supply during times of scarcity. The mathematical equation for this is defined as:

(19) $$X^{i}\left(t+1\right)=\left\{\begin{array}{l} X^{i}\left(t\right)\;\;\;\;if\;{\;fitness}_{old}^{i}>{fitness}_{new}^{i}\\ X^{i}\left(t+1\right)\;\;\;\;\;\;\;\;\;\;\;\;\;\;\;\;\;\;\;\;\;\;\;else \end{array}\right.$$

where ${fitness}_{old}^{i}$ and ${fitness}_{new}^{i}$ are fitness values before and after updating the location of the ith red-billed blue magpie, respectively.

### 3.3. An Improved Multi-Objective Red-Billed Blue Magpies Optimizer

**Figure 4:**
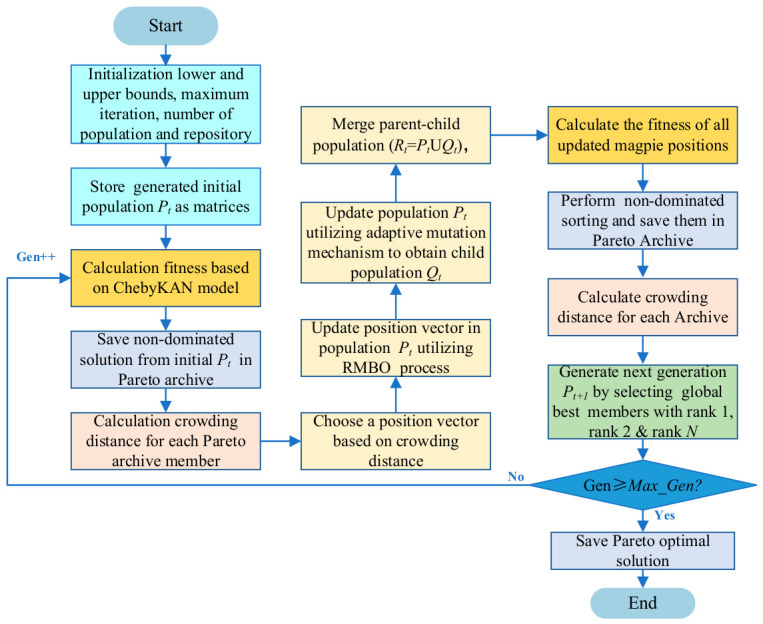
Flowchart of IMORBMO using an adaptive mutation mechanism based on the information entropy.

This subsection presents an improved, Red-billed Blue Magpie Optimizer (IMORBMO), which integrates the Chebyshev KAN surrogate model to improve both robustness and computational efficiency in high-dimensional aerodynamic design optimization. Based on the search mechanism of the original RBMO, the proposed IMORBMO extends the framework to handle multi-objective problems that integrate four core components: non-dominated sorting, crowding distance estimation, a search mechanism adapted from the single-objective Red-billed Blue Magpie Optimizer (RBMO), and an adaptive mutation operator based on information entropy. The overall workflow of IMORBMO is illustrated in Figure 4.

Step 1: Initialization parameters

This IMORBMO metaheuristic algorithm starts with the initial parameters like lower and upper bounds of agents, maximum number of iterations, number of populations, and number of repositories. Then, an arbitrarily formed population *P_t_* (equivalent to the red-billed blue magpie’s position of the agent) with feasible search space *S* is generated.

Step 2: Non-dominated sorting and saved solution in Pareto Archive

Calculation of the fitness functions in the space vector for each member of *P_t_* (*the subscript t =* 0) based on the Chebyshev KAN surrogate model. The Magpie fitness functions are sorted using the concept of non-dominated sorting. Next, identify the first rank by selecting non-dominated individuals from the initial population, which are assigned to the first non-domination rank(rank-1). These top-ranked individuals are assigned to the first front and excluded from the original population. Additionally, the non-dominated individuals in the remaining populations are allocated the second rank and positioned in the second front. This process persists until every individual is categorized into distinct fronts according to their ranks.

Step 3: Crowding distance evaluation

This stage focuses on calculating the crowding distance (*CD*) to assess the distribution density of feasible solutions near a specific point. The *CD* represents the average distance between two solutions on either side of a given point. When comparing solutions with varying *CD*, the one exhibiting a greater distance is situated in a less densely populated area. The *CD* of the *ith* individual is determined by averaging the distance to its nearest neighbors on either side, as specified as follows:

(20) $$CD\left(i\right)=\sum_{i=1}^{m}\frac{f_{j}^{i+1}-f_{j}^{i-1}}{f_{j}^{max}-f_{j}^{min}}$$

where m is the number of objective functions, $f_{j}^{max}$ and $f_{j}^{min}$ represent the maximum and minimum values of the jth objective function.

The stage consists of the optimal positions of the search agent guiding the global optimum and removing the ones with the lowest crowding distance based on the archive size.

Step 4: Update population *P_t_* with the RBMO algorithm and an adaptive mutation mechanism

This phase updates the current population *P_t_* through application of the RBMO algorithm and an adaptive mutation mechanism based on information entropy. Update position vector of the red-billed blue magpie utilizing RBMO process Equations (15)–(19). Subsequently, update population *P_t_* again using the adaptive mutation mechanism to generate a new kid population *Q_t_*. The mathematical formulation of the adaptive mutation mechanism is as follows:

An information entropy of the population $H_{global}$ is defined as:

(21) $$\left\{\begin{array}{c} H_{global}=-\sum_{i=1}^{k}p_{i}\log p_{i}\\ p_{i}=d_{i}/\sum_{j=1}^{k}d_{j}\;\;\;\;\;\;\;\;\;\;\;\;\;\;\;\; \end{array}\right.$$

where $p_{i}$ is the information entropy of each individual position, and $d_{i}$ is the average distance from the *i*th solution to its nearest neighbors.

The local information $H\left(j\right)$ reflects the uniformity of the distribution of surrounding neighbors. The higher the value of $H\left(j\right)$, more uniform is the distribution around the solution, indicating greater diversity in the region of solution sets. The mathematical expression is defined as:

(22) $$H\left(j\right)=-\sum_{i=1}^{k}p_{ij}logp_{ij}$$

where $p_{ij}$ is the proportion distance between the *j*th solution and its *i*th nearest neighbor.

The comprehensive selection probability is given as

(23) $$P\left(j\right)=\frac{1}{R(j)}\cdot \frac{H(j)}{H_{total}}$$

where R(j) is the Pareto rank of the jth Pareto solution, the larger $P\left(j\right)\;$ with a smaller rank, H(j) is the local information entropy of the jth Pareto solution based on the k-nearest neighbors, and $H_{total}$ is the sum of information entropy across all solutions.

This mechanism enables solutions with low rank and high diversity to achieve better accuracy. The mutation strength is adjusted based on the global information entropy $H_{global}$ of the population.

(24) $$\vartheta \left(H_{global}\right)=\left\{\begin{array}{c} 0.5\cdot \exp\left(-\frac{H_{global}}{H_{0}}\right)\;\;{if\;H}_{global}<H_{0}\\ 0.01+0.1\cdot (1-\frac{H_{global-H_{0}}}{H_{max}-H_{0}})\;otherwise\; \end{array}\right.$$

where ϑ=0.5, higher mutation intensity is applied to explore new regions when solutions are densely distributed, and smaller mutation intensity is used when solutions are sparsely distributed to maintain convergence stability.

The updated position of the search agent after mutation is defined as follows:

(25) $$W_{i}^{mut}=W_{i}+\;\vartheta .∆.N(0,\Sigma )$$

where $∆\;=diag\left(U-L\right)$ is a diagonal matrix of the search range, Σ is an adaptive covariance matrix adjusted according to the population distribution, $W_{i}\;$ is each individual position of the search agent.

Next, integrating *P*_t_ and *Q*_t_ to obtain a new population *R_t_* is done, upon which the non-dominated sorting technique is repeatedly executed on *R_t_*. Then, the members of *R_t_* are ranked through non-domination levels in various categories.

Step 5: Generate the next population *P*_t+1_

The next process involves selecting *N* elite members from *R_t_* to create the next population *P*_t+1_. When the first rank’s size exceeds or matches *N*, the IMORBMO selects *N* members from the least crowded area to constitute *P*_t+1_. Conversely, if the first rank has fewer than *N* members, all individuals from this front are directly incorporated into *P*_t+1_, and additional candidates are sourced from the least crowded region of the second rank. If *P*_t+1_. remains undersized, the same strategy is iteratively applied to subsequent ranks until the population reaches the required size of *N*.

Step 6: Output Pareto-optimal solutions

The algorithm evaluates the termination criterion by comparing the current number Gen with the predefined maximum number of iterations. If the iteration number Gen is less than max iteration number, the generation counter is incremented (Gen = Gen + 1), and the process resumes from the second phase, iteratively generating successive populations *P*_t+2_, *P*_t+3_, *P*_t+4_, … etc. The loop continues until the $Gen\gg {Gen}_{max}$, the algorithm terminates and returns the final set of non-dominated Pareto-optimal solutions.

### 3.4. Evaluation Metrics for MOWMA

MO optimizers typically generate Pareto fronts consisting of multiple non-dominated solutions. The resulting Pareto fronts often differ across various algorithms. To assess the performance of MOWMA, several metrics have been identified: generational distance (GD) evaluates the algorithm’s efficiency and reliability. Inverted generational difference (IGD) quantifies the convergence and diversity of the solution set. Spacing (SP) measures the uniformity and spread of solutions, while the hypervolume indicator (HV) provides a robust metric for analyzing both diversity and convergence in algorithmic outputs.

(26) $$\begin{array}{l} GD=\sqrt{\sum_{i=1}^{no}d_{i}^{2}}/n\\ d_{i}=\min_{j}\left(\left|f_{1}^{i}(\vec{x})-f_{1}^{j}(\vec{x})\right|+\left|f_{2}^{i}(\vec{x})-f_{2}^{j}(\vec{x})\right|\right) \end{array}$$

(27) $$IGD=\sqrt{\sum_{i=1}^{nt}{\left({d^{\prime }}_{i}\right)}^{2}}/n$$

(28) $$SP=\sqrt{\frac{1}{n-1}\sum_{i=1}^{n}{\left(\bar{d}-d_{i}\right)}^{2}}$$

(29) $$\mathrm{HV}=\Lambda \left(\underset{s\in PF}{\cup }\left\{s^{\prime }|s≺s^{\prime }≺s^{nadir}\right\}\right)$$

where *no* represents the number of true Pareto solutions, *nt* denotes the number of true Pareto-optimal solutions, $\bar{d}$ is the average of all $d_{i}$, ${d^{\prime }}_{i}$ specifies the Euclidean distance, and *n* is the number of obtained Pareto fronts.

## 4. Data Acquisition Experiment

**Figure 5:**
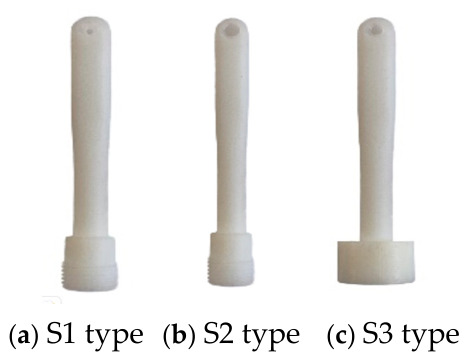
3D printing models of the relay nozzles.

Recent advances in additive manufacturing, particularly 3D printing, have significantly expanded its applicability in the fabrication of complex functional materials and components. Unlike conventional manufacturing techniques, which often require costly tooling and are limited in geometric freedom, 3D printing offers exceptional design flexibility, enabling the rapid, mold-free production of intricate structures with high reproducibility and customization. We manufacture a series of relay nozzles with distinct internal geometries using a high-precision 3D printing technique. These representative 3D printing models, S1, S2, and S3, were presented in Figure 5.

**Figure 6:**
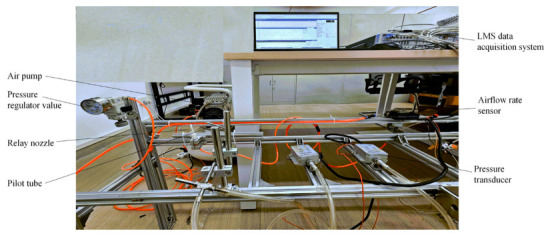
Airflow speed and air flow rate acquisition system.

The experimental apparatus comprises a Siemens LMS data acquisition system (SCADAS, Siemens, Munich, Germany), a MEMS flow rate sensor (model AFM0725H00S, Asair, Guangzhou, China), a pitot tube, two pressure transducers, relay nozzles, a pressure regulator valve, and an air pump. The software Simcenter Testlab 2021.2. is used for data acquisition. All experimental apparatus is depicted in Figure 6. The relay nozzles were supplied with high-pressure air by a pneumatic pump. The inlet pressure was regulated using a pressure control value to ensure an operating pressure of 0.3 MPa. A MEMS mass flow sensor was mounted at the relay nozzle exit to guarantee high precision airflow rate during real-time data acquisition. The flow rate ranges from 0 to 50 L/min. Additionally, a pilot tube is mounted 15 mm from the exit of the relay nozzle. The airflow velocity range of the pilot tube ranges from 0 to 340 m/s. The dynamic and static pressure of the pilot tube were measured with pressure transducers, respectively. The voltage output range of all sensors is between 0.5 V and 4.5 V. The Siemens LMS data acquisition system collects information from airflow rate and pressure sensors, which convert analog voltage signals into digital signals.

## 5. Results and Discussion

### 5.1. Data Description

To obtain the mapping relationship between the structural parameters and the first objective (airflow speed) and the second objective (air consumption), the Latin Hypercube sampling (LHS) method was adopted to design samples, including 120 groups of auxiliary nozzles. The dataset was split into training, validation and test sets in a ratio of 7:3. Among them, the value range of inlet diameter *D*1 is [6, 12.0], straight pipe diameter *D*2 is [4, 10.0], exit diameter *d* is [0.8, 1.8], and the value range of *α* is [10°, 80°]. Table 1 lists the 10 groups of airflow speed (*V*) and air consumption (*Q*) data of various auxiliary nozzles.

As presented in Table 1, a comparative analysis of various auxiliary nozzles indicates that the airflow rate increases dramatically from 3.21 L/min to 27.38 L/min. An in-depth analysis of the airflow rates in each group indicates an obvious impact of the change in outlet diameter on the airflow rate. Especially, the airflow rate varies from 4.08 L/min to 10.36 L/min for the No.3 and No.4 relay nozzles. The variance in airflow rate between No.3 and No.4 auxiliary nozzles reveals that the outlet diameter of the relay nozzle is a critical parameter affecting the airflow rate. The airflow velocity increases dramatically from 92.9 m/s to 232.8 m/s for the No.2 relay nozzle and the No.9 relay nozzle. It illustrates the strong nonlinear relationship between the airflow velocity and high-dimensional structural parameters of the relay nozzle.

### 5.2. Optimization Hyperparameter of the Chebyshev KAN Model

All prediction tasks in this study were conducted on a desktop computer running the Windows 10 operating system. This device is equipped with an AMD EPYC 7702 64-Core Processor, 64 GB of RAM and an NVIDIA GeForce RTX 4080 SUPER GPU, providing substantial computational power for deep learning workloads. The surrogate model was implemented in Python 3.9.13 using the PyCharm 2025.1 integrated development environment. PyTorch 12.6 served as the primary deep learning framework for model development and training, while Keras was employed for implementing baseline neural network models for comparison. Each regression task was completed by the surrogate model in under 300 s, demonstrating its feasibility for real-time applications. To ensure numerical stability during training, all input features were normalized to the range [0, 1] using the Min-Max scaling method prior to training various surrogate models. This preprocessing step mitigates the adverse effects of widely varying feature scales, which can otherwise lead to unstable gradient updates and hinder convergence.

In this paper, four surrogate models—MLP, CNN, standard KAN and the proposed Chebyshev KAN were compared under the same evaluation conditions. The CNN model consists of two convolutional layers with a kernel size of two, containing 64 and 32 filters, respectively. It is trained for 100 epochs using the Adam optimizer with a learning rate of 0.01, a mini-batch size of 32, and a dropout rate of 0.2. The MLP model features an input dimension of four, followed by two shared fully connected layers with 64 neurons each. Each output task is handled by a dedicated head comprising two additional fully connected layers with 32 and one neuron(s), respectively. The GELU activation function is employed throughout. Training spans 200 epochs with Adam (learning rate: 3 × 10^−4^), batch size 32, and a dropout rate of 0.25. To enhance stability and generalization, layer normalization and gradient clipping are applied as regularization techniques.

Furthermore, the standard KAN model uses an input dimension of four and a hidden dimension width of 16, without a task-specific output head. It employs the SiLU activation function and is optimized with Adam(learning rate: 1 × 10^−3^) over 100 epochs, using a mini-batch size of 32. The proposed Chebyshev KAN shares the same training data and preprocessing, replacing the original B-spline activations with Chebyshev polynomial expansions, aiming to improve approximation accuracy—particularly under limited data regimes.

**Figure 7:**
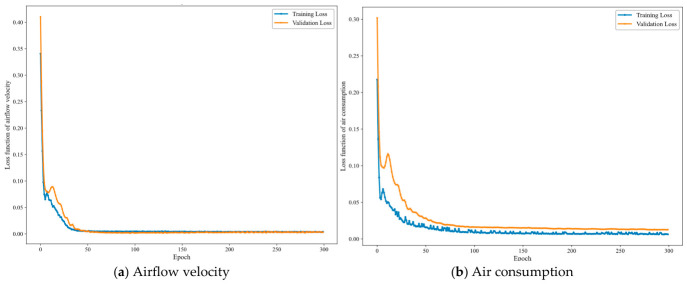
Loss function with iterations for airflow velocity and air consumption.

Figure 7 shows the convergence curves of the loss function corresponding to each iterative process for airflow velocity and air consumption. As seen from Figure 7a, the loss function associated with airflow velocity begins to converge steadily around the 40th iteration and reaches its minimal fitness value by the 50th iteration. Similarly, as shown in Figure 7b, the loss function for air consumption starts converging at approximately the 50th iteration and attains its optimal fitness value at the 80th iteration. Although the convergence for air consumption requires more iterations, the consistent downward trend without divergence underscores the robustness and reliability of the hyperparameter tuning procedure employed in the Chebyshev KAN surrogate model. Collectively, these results validate the effectiveness of the optimization framework in achieving both convergence stability and high-fidelity surrogate modeling across multiple output responses.

### 5.3. Comparative Experiment Analysis

The accuracy of the Chebyshev KAN surrogate models for predicting airflow velocity and air consumption was quantitatively assessed using three statistical metrics, including mean absolute error *(MAE)*, root mean square error *(RMSE)* and the coefficient of determination *(R*^2^*)*. These metrics provide a thorough assessment of performance in terms of peak error, average error, and overall correlation with the predicted data, respectively.

Table 2 lists three evaluation indicators of airflow velocity and air consumption in the test set. The conventional MLP model exhibits relatively poor predictive capability, with MAE 10.221 for airflow velocity and 3.502 for air consumption. Similarly, the CNN model yields substantial prediction errors, reporting MAE values of 8.856 and 3.348 for the two objectives, respectively. Furthermore, the Kolmogorov–Arnold Network (KAN) achieves an MAE of 8.431 for airflow velocity and 2.111 for air consumption, respectively. In contrast, the proposed Chebyshev KAN surrogate models achieve remarkable accuracy. The *MAE* values for the airflow velocity and air consumption are 0.103 and 0.115, respectively. The *RMSE* of Chebyshev KAN surrogate models is 0.047 for airflow velocity and 0.056 for air consumption, respectively. Additionally, the coefficient of determination (*R*^2^) values of 0.975 and 0.950 for the two objectives are very close to one. These results confirm the superior predictive accuracy and robustness of the proposed Chebyshev KAN surrogate models for multi-output aerodynamic response prediction.

Conventional deep learning models (MLP and CNN) exhibit high computational overhead, requiring 20.294 to 24.970 s for training and 2.538 to 3.013 ms/sample for inference. Standard KAN requires 11.565 s and 2.083 ms/sample, respectively. Among traditional models (Kriging, SVR, and RBF), Kriging performs best overall, offering balanced predictive accuracy and relatively fast inference (2.081 ms/sample); however, this group as a whole still requires 10.461 to 14.852 s to train and 2.081 to 2.450 ms/sample to infer. In contrast, the proposed Chebyshev KAN achieves remarkable superiority, demanding the shortest training time (9.495 s) and lowest inference latency (1.370 ms/sample). Under the same evaluation budget, the estimated end-to-end speedup over CFD reaches $2.1278\times 10^{4}\;s$, indicating a substantial reduction in computational cost with preserved predictive quality. This confirms its exceptionally lightweight architecture, providing the rapid evaluation speeds critical for high-dimensional multi-objective aerodynamic optimization without compromising accuracy.

**Figure 8:**
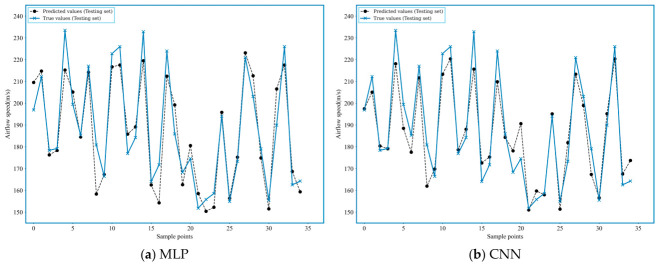

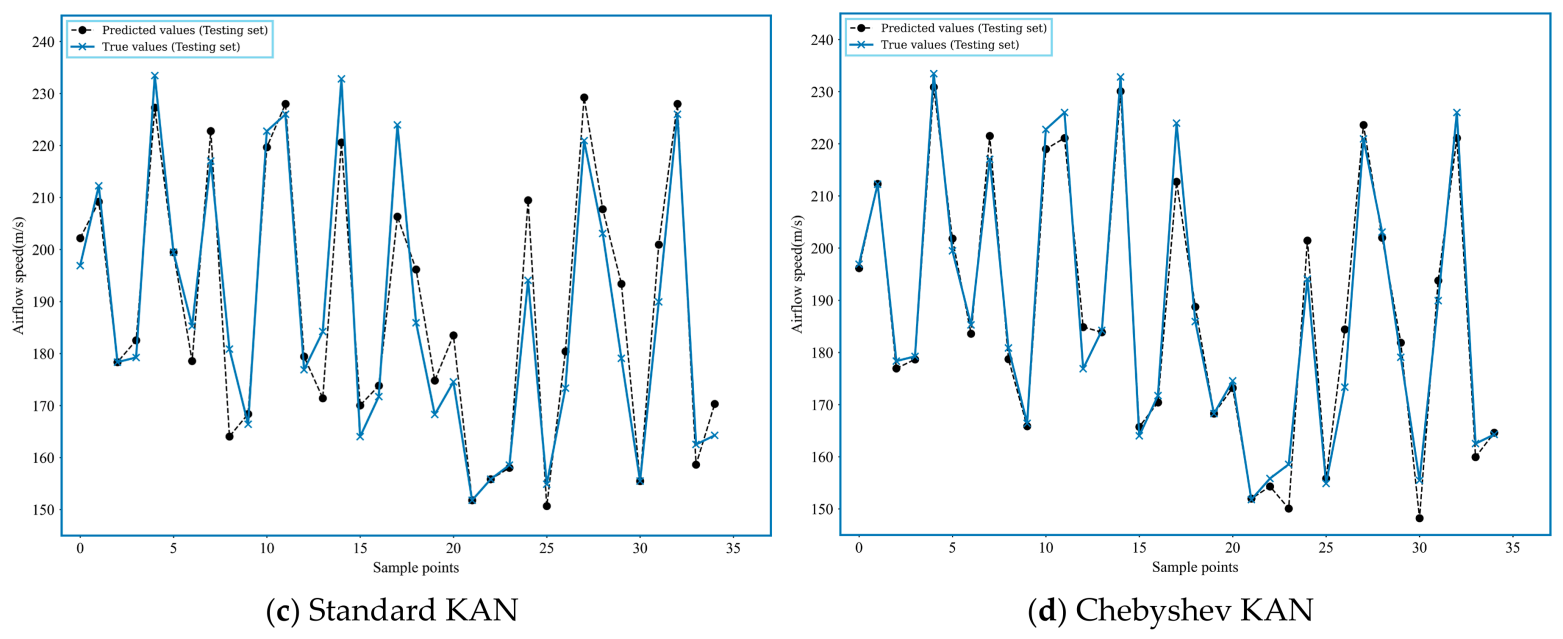
Prediction results of airflow velocity using various models.

Figure 8 illustrates a comparative line graph of the predicted versus actual airflow velocity obtained by four surrogate models: MLP, CNN, standard KAN, and Chebyshev KAN. The deviation between the predicted values and actual values can be clearly distinguished among various models. As shown in Figure 8a, the MLP model exhibits significant deviations between its predictions and actual values with the experimental apparatus, particularly in regions with sharp velocity gradients. This suggests that the MLP struggles to generalize well to prediction accuracy under limited training data. As seen in Figure 8b, the CNN model demonstrates improved prediction accuracy but still suffers from relatively large fluctuation amplitudes at peak velocity. Figure 8c illustrates the standard KAN model that produces obvious discrepancies in velocity valley regions. These biases suggest the B-spline series introduces insufficient resolution in regions of high-velocity gradient, leading to underfitting. Figure 8d illustrates that the Chebyshev KAN model achieves markedly superior alignment between predicted and actual airflow velocities. The deviation is minimal across the entire domain, with near-perfect tracking of both peak and valley structures. This high fidelity stems from the replacement of B-spline activations with Chebyshev polynomial expansions, which offer several critical advantages: suppression of Runge’s phenomenon—by clustering basis function nodes near interval endpoints, they effectively mitigate oscillatory behavior commonly observed with high-degree polynomial interpolation.

**Figure 9:**
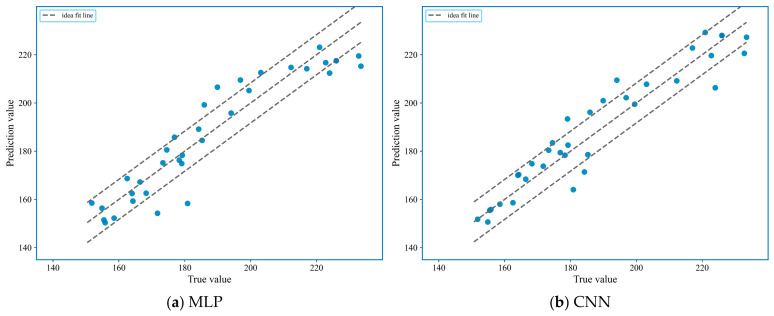

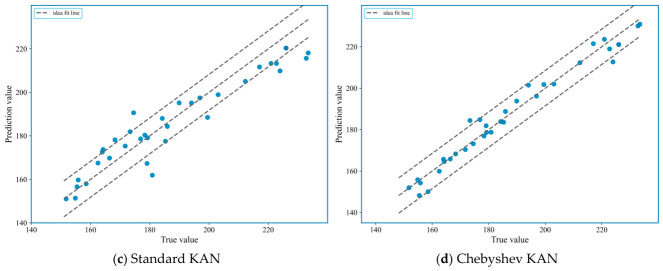
Regression plot of airflow velocity using various models.

Figure 9 presents the regression scatter plot of predicted airflow velocity values on the test set for the MLP, CNN, original KAN, and the proposed Chebyshev KAN models. In each subplot in Figure 9, the diagonal line represents perfect prediction, while the upper and lower dashed lines delimit an acceptable error margin of ±8 m/s around the true airflow velocity. As shown in Figure 9a,b, the scatter points of both the MLP and CNN models exhibit substantial deviations from the diagonal, with a significant portion of predictions falling outside the ±8 m/s error band. This indicates limited modeling capacity and poor generalization performance for these deep learning architectures in capturing complex, high-dimensional features, and multiple objectives. As seen in Figure 9c, the majority of KAN predictions cluster more closely around the diagonal, suggesting enhanced fitting capability and better prediction accuracy compared to MLP and CNN. In Figure 9d, the most compelling performance is observed by the Chebyshev KAN models. Here, the scatter points are even more tightly concentrated along the diagonal, with nearly all predictions residing well within the prescribed error tolerance. This demonstrates not only superior regression accuracy but also exceptional robustness and generalization, particularly given the relatively small size of the training dataset. The results consistently affirm that Chebyshev KAN achieves the best balance among fitting precision, error control, and generalization ability. The underlying reason for this superior performance lies in the expressive power of the Chebyshev polynomial employed in Chebyshev KAN as the activation function. Unlike fixed nonlinearities in traditional neural networks or even the spline series in standard KAN, Chebyshev expansions provide a spectrally orthogonal and numerically stable basis for approximating high-dimensional, nonlinear mappings.

**Figure 10:**
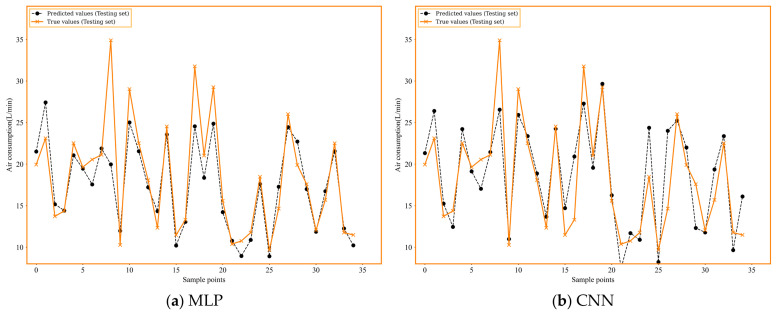

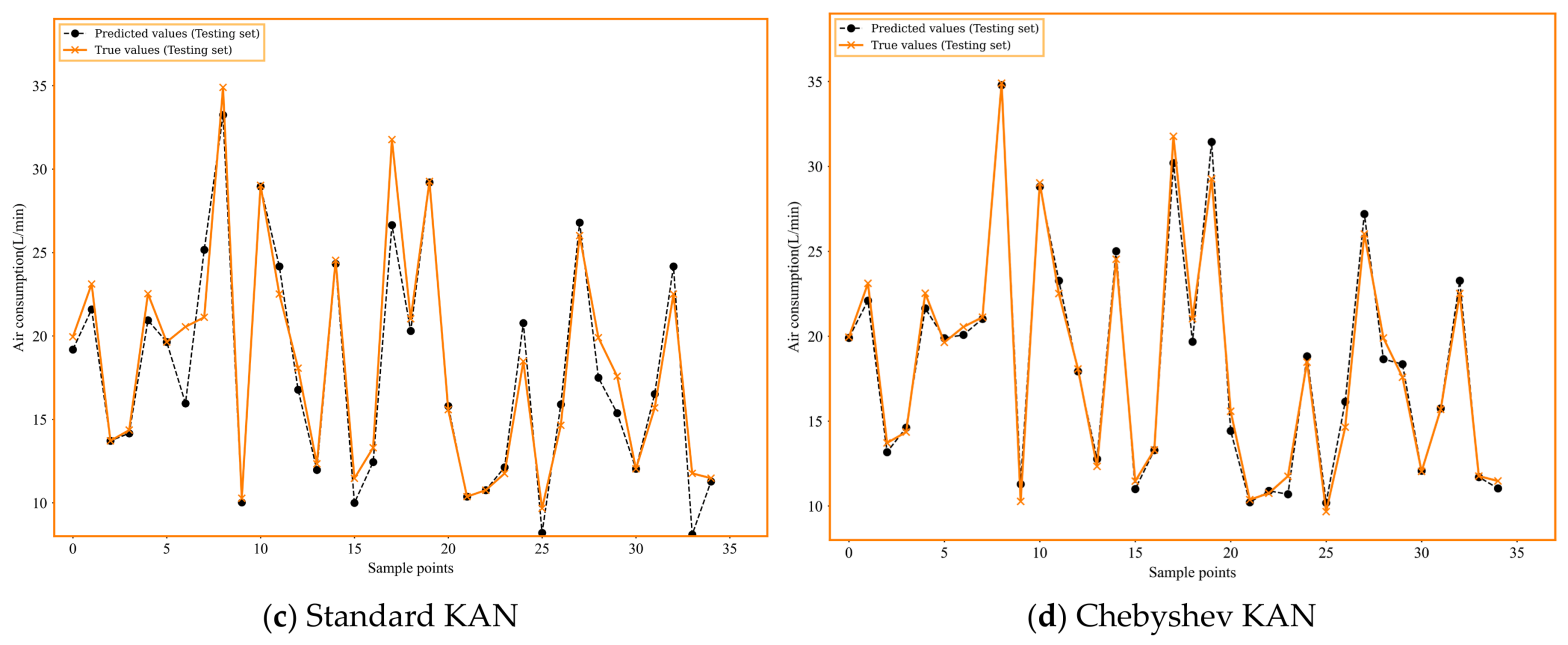
Prediction results of air consumption using different surrogate models.

Figure 10 presents the line plots comparing the predicted and actual air consumption values for MLP, CNN, standard KAN, and Chebyshev KAN models. As shown in Figure 10a, the MLP model exhibits substantial prediction errors on the test set. Its output frequently diverges from the true values, accompanied by fluctuations that indicate poor stability and capacity to fit high-dimensional input features and air consumption response with a limited-size training dataset. As seen in Figure 10b, the CNN model has the ability to extract local spatial features. However, there are obvious discrepancies between the prediction peak value and the actual values of the CNN model. As seen in Figure 10c, the standard KAN model demonstrates improved performance relative to MLP and CNN, but still suffers from inaccuracies. In particular, several predicted peak values deviate significantly from their true values. The errors suggest that KAN’s learnable activation functions enhance expressivity over fixed nonlinearities; they may still lack sufficient regularization to fully capture sharp transitions on the peak values. In contrast, as shown in Figure 10d, the Chebyshev KAN model achieves remarkable alignment between predicted and actual values of air consumption. Both peak and valley values are accurately tracked with minimal amplitude distortion, indicating strong fitting capabilities and high precision. The superior performance underscores Chebyshev KAN model enhanced representational ability, by leveraging orthogonal basis functions with favorable approximation properties. Thereby, the Chebyshev KAN can efficiently capture intricate structural features and nonlinear dependence with limited training data.

**Figure 11:**
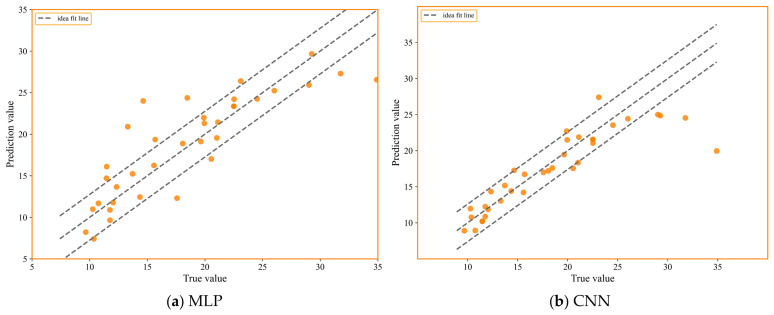

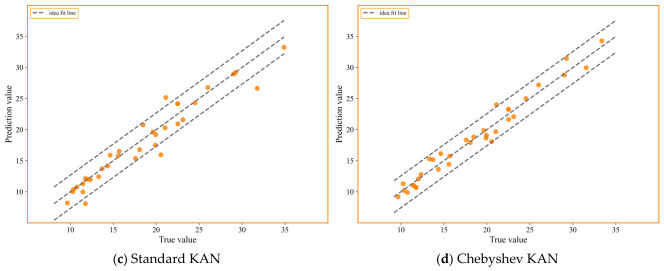
Regression plot of air consumption using various models.

Figure 11 shows the regression scatter plots of prediction and actual air consumption values on the test set using MLP, CNN, standard KAN, and Chebyshev KAN models. The regression scatter illustrates the fitting ability of various models for high-dimensional nonlinear input features and outputs. In each subplot in Figure 11, the diagonal line represents perfect prediction, while the upper and lower dashed lines delimit an acceptable error margin of ±2 L/min around the true air consumption. As shown in Figure 11a, most predicted points of the MLP model are significantly deviated from the diagonal, with dispersed density, and *RMSE* exceeds 3.502, and the determination coefficient *R*^2^ for MLP is only 0.72, indicating poor prediction performance. As shown in Figure 11b, the scatter points of CNN models further concentrate towards the diagonal, with *RMSE* lower than 2.046. As shown in Figure 11c, the determination coefficient *R*^2^ for KAN exceeds 0.907, notably higher than that of MLP and CNN. As seen in Figure 11d, the Chebyshev KAN model shows a high degree of linear correlation between the predicted values and the actual values, with the scatter points highly concentrated near the diagonal, especially in the middle range, where the fitting effect is the most significant. The determination coefficient of Chebyshev KAN is *R*^2^ = 0.950, indicating that the overall predicted values are along with actual values, and the deviation degree is small; *RMSE* = 0.056, further verifying its strong advantage in error control and demonstrating extremely low prediction errors. This study systematically compared several mainstream models in prediction air consumption of the relay nozzle. The results confirm that the Chebyshev KAN surrogate model has advantages in nonlinear high-dimensional MO regression tasks, offering controlled approximation errors.

**Figure 12:**
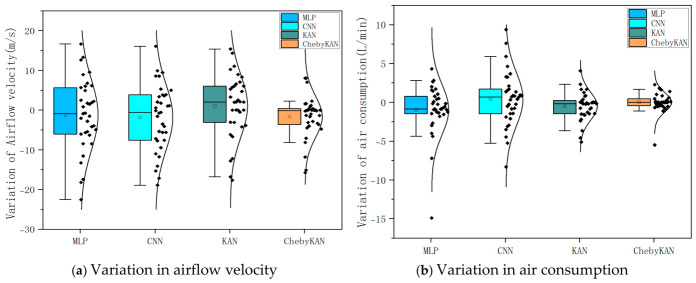
Normal distribution and standard deviation of airflow velocity and air consumption using different models.

Figure 12 presents the prediction errors for airflow velocity and air consumption across different surrogate models. In each subplot, the vertical bars denote the standard deviation (SD) of the predictions, while scattered points illustrate the residuals between the actual values and the predicted values on the test set. In the Figure 12, the squares represent the arithmetic mean of predicted data in the testing data set. The horizontal line within the box represents the median of the predicted data. The upper and lower boundaries of the box represent the upper quartile and the lower quartile respectively. The height of the box denotes the interquartile range (IQR).Whiskers typically represent the data range. A statistical evaluation of the prediction results in testing sets, highlighting the uncertainties across different models. For the airflow velocity as shown in Figure 12a, the scattered points of all models follow a normal distribution. The maximum absolute deviations of airflow velocity are 22.52 m/s, 17.1 m/s, 16.8 m/s and 15.8 m/s for the MLP, CNN, standard KAN, and Chebyshev KAN models, respectively. Correspondingly, their standard deviations are 8.970, 8.266, 7.628, and 5.012 for the MLP, CNN, standard KAN, and Chebyshev KAN models, respectively. Notably, the residuals of the Chebyshev KAN model are more tightly concentrated around the mean, indicating significantly lower uncertainty and higher fidelity in predicting airflow velocity. Regarding air consumption as shown in Figure 12b, the standard deviation values for MLP, CNN, standard KAN, and Chebyshev KAN models are 3.259, 3.162, 1.786, and 1.112, respectively. Chebyshev KAN surrogate model achieves the narrowest error band for air consumption, underscoring its superior predictive accuracy. This improvement stems from its use of Chebyshev polynomial expansions, which enhance approximation capability while mitigating overfitting, thereby yielding more precise and robust predictions.

### 5.4. Results on ZDT and WFG Functions from CEC 2020

To evaluate the effectiveness of the Improved Multi-objective Red-billed Blue Magpie Optimizer (IMORBMO) in addressing real-world engineering application challenges, the proposed IMORBMO was tested on a suite of widely recognized benchmark problems from the CEC 2020 [27]. Among these, the ZDT and WFG were selected due to the ability to represent a broad spectrum of multi-objective scenarios ranging from simple convex fronts to non-convex, discontinuous, and deceptive landscapes. These benchmark functions are bi-objective, nonlinear, and feature well-defined yet diverse Pareto-optimal fronts, making these functions ideal for assessing both convergence accuracy and solution diversity. Specific details can be found in the relevant literature [28].

The performance of the proposed IMORBMO was evaluated against five state-of-the-art multi-objective metaheuristics: MOEAD [21], MOWOA [22], MODBO [23], MODA [24], and MORBMO [25]. To ensure a fair and comprehensive comparison, six multi-objective algorithms were tested with identical settings: a population size of 100, a maximum of 500 iterations, and an external archive(repository) capacity of 100 solutions. Accounting for the inherent stochasticity of metaheuristic algorithms, each algorithm was executed independently over 20 runs to yield statistically reliable results. The mean value (*f_mean_*) and standard deviation(*f_std_*) were adopted as the primary indicators, as commonly used for assessing consistency and robustness.

Table 3 illustrates the statistical results of the inverted generational distance (IGD) metric for the proposed IMORBMO algorithm in comparison with MOEAD, MOWOA, MODBO, MODA, and MORBMO algorithms. On ZDT1, IMORBMO achieves the optimal performance, yielding a mean IGD value(*f_mean_*) of 2.746 × 10^−3^ and the lowest standard error(*f_std_*) of 1.581 × 10^−3^, indicating high accuracy. On ZDT2 and ZDT3 functions, IMORBMO achieves the smallest mean IGD value(*f_mean_*) of 3.452 × 10^−3^ and 3.393 × 10^−3^, respectively. The IGD metrics of IMORBMO are basically better than those of other algorithms. The IGD results demonstrate that IMORBMO delivers superior and competitive performance across the tested benchmark functions. This reflects its enhanced ability to balance convergence toward the true Pareto front and maintain solution diversity. Notably, on WFG2, WFG3 and WFG4 test problems, IMORBMO also obtains the lowest mean IGD value along with the smallest standard deviation, excellent in handling nonlinearity, bias, and degenerate Pareto fronts. Such performance is particularly valuable in real-world applications such as aerodynamic design optimization, where problems involve a high-dimensional nonlinear search space and multiple conflicting objects.

Table 4 illustrates the hypervolume (HV) metric results derived from all compared MO algorithms on ZDT and WFG benchmark suites. The HV indicator provides a unified measure of both convergence and solution diversity. A large HV value usually means that the algorithm can better approximate and cover the true PF, indicating not only proximity to the true Pareto front but also broad convergence across objective space. IMORBMO achieves the highest mean HV metric on ZDT1, ZDT2 and ZDT3, demonstrating superior performance in capturing both convex and non-convex PF geometrics. Moreover, it exhibits consistently low standard deviations, indicating high solution stability across independent runs. It is clear that the proposed IMORBMO effectively balances convergence and solution diversity.

On the WFG2-WFG4 test problem, IMORBMO also outperforms the above-mentioned algorithms in terms of HV, further validating its capability to handle scalable, high-dimensional, and structurally complex landscapes. This consistent advantage stems from its integration of an adaptive mutation mechanism with the search strategies of the RBMO, inspired by the red-billed blue magpie’s foraging behaviors. This adaptive mutation strategy dynamically adjusts exploration intensity based on population distribution and evolutionary progress, enhancing local exploitation near the Pareto front.

Table 5 illustrates the generational distance (GD) results obtained by all compared MO algorithms on the ZDT and WFG benchmark suites. On ZDT1, IMORBMO achieves the lowest mean GD value of 4.091 × 10^−3^, accompanied by the smallest standard error of 2.081 × 10^−4^. On ZDT2 and ZDT3, IMORBMO attains the smallest mean GD value of 4.161 × 10^−3^ and 1.561 × 10^−3^, respectively. In WFG cases, IMORBMO also has the mean GD value 1.624 × 10^−3^, 1.321 × 10^−4^ and 5.225 × 10^−3^ for WFG2, WFG3, and WFG4, respectively. The standard deviations of IMORBMO are consistently lower than those of the MOEAD, MOWOA, MODBO, MODA and MORBMO. These results highlight the effectiveness of adaptive mutation strategies based on information entropy. This superior performance stems from IMORBMO’s dynamic selection probability that prioritizes individuals with low rank and strong diversity contributions. Moreover, this dynamic mutation scheme enhances population exploration while escaping local optima. In contrast, the original MORBMO is prone to premature convergence and suboptimal solutions. It is evident that the IMORBMO outperformed the mentioned MO algorithms with superior trade-off balance and Pareto-optimal sets exploration ability.

**Figure 13:**
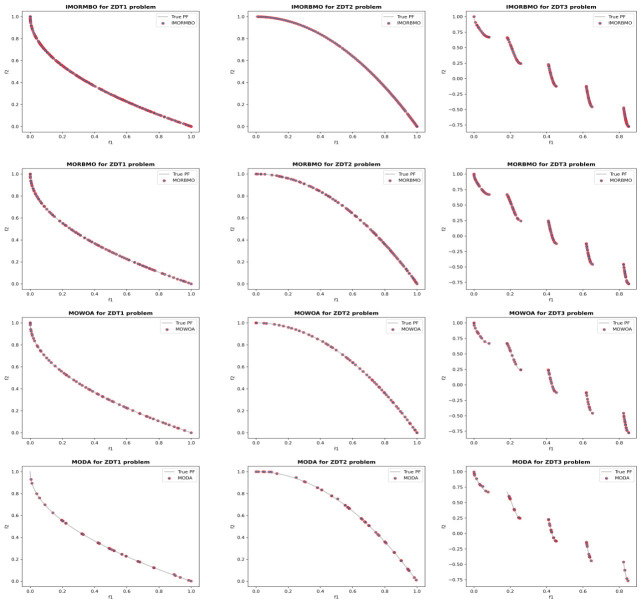
Pareto fronts with various algorithms on the ZDT function.

Figure 13 shows the best Pareto front of all considered MO algorithms on the ZDT test functions. ZDT1 represents a convex Pareto front, and ZDT2 is a non-convex front. As seen in Figure 13, the ZDT1 function is characterized by a concentration of points within a small range close to the boundary of $f_{2}\left(x\right)=0$, and most of them are out of the correct lower bound $f_{1}\left(x\right)=0$. On the ZDT1 function, the IMORBMO achieves a closer convergence to the curves of true Pareto fronts compared to other algorithms. On ZDT2, the IMORBMO exhibits superior uniformity and accuracy across the entire Pareto front. The Pareto front obtained by the IMORBMO is maximized for each target, with a broad spectrum of values covered by non-dominated solutions. On the disconnected function ZDT3, the IMORBMO also performs the strongest convergence performance, producing uniformly distributed solutions and the highest accuracy along the true Pareto fronts. However, the Pareto front was disrupted by the MORBMO, MOWOA and MODA, and was sparse in some regions on the ZDT2. Moreover, MODA has difficulties evolving well-distributed trade-off fronts on the ZDT1, ZDT2 and ZDT3 functions.

**Figure 14:**
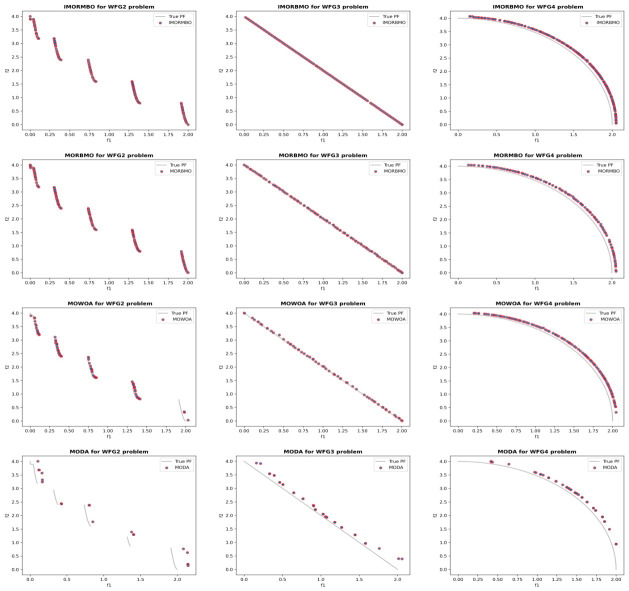
Pareto fronts with various algorithms on the WFG function.

Figure 14 presents the Pareto-front approximations obtained by the compared algorithms on representative WFG instances- namely, WFG2, WFG3 and WFG4. The WFT test function is intended to assess the MO algorithm′s capability to solve highly intricate and nonlinear problems. As shown in Figure 14, the IMORBMO demonstrates superior performance across the WFG suite, which is designed to evaluate algorithms under highly nonlinear and geometrically complex Pareto fronts. On WFG2, the Pareto front exhibits oscillatory characteristics. Particularly, the IMORBMO closely follows the overall shape of the true value with significant deviations that other compared MO algorithms, indicating enhanced convergence. On WFG3, the solutions produced by IMORBMO nearly coincide with the true Pareto front, reflecting high accuracy. On WFG4, the IMORBMO produces smooth and accurate solutions along the true Pareto front, maintaining a uniform solution distribution. In contrast, IMORBMO yields a solution set with inadequate diversity and poor uniformity on the WFG3 and WFG4. MOWOA also exhibits insufficient diversity and uniformity on the WFG3 and WFG4, and the MODA algorithms show noticeable deviations on the WFG2 and WFG3 functions.

These results suggest that IMORBMO is effective in handling irregular and shape-complex Pareto fronts, with a notable ability in preventing solution dispersion away from the target manifold. Moreover, the IMORBMO achieves a favorable balance between convergence and diversity in optimizing multiple conflicting objectives. This superior performance can be attributed to its adaptive mutation mechanism, which dynamically updates the population to enhance global exploration while avoiding getting trapped in local optima. Consequently, IMORBMO effectively preserves solution diversity and yields the closest approximation to the true Pareto front among all compared approaches.

### 5.5. Optimization of the Aerodynamic Performance of Relay Nozzle

This subsection presents an application of IMORBMO integrating a Chebyshev KAN surrogate model for aerodynamic design of the relay nozzle. In consideration of the conflict between two objective functions, the design must strike a compromise between maximum airflow velocity and minimum air consumption of the relay nozzle.

**Figure 15:**
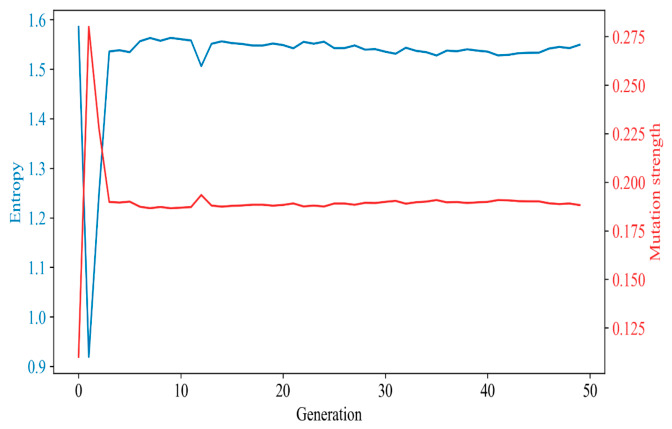
Entropy–mutation co-evolution in IMORBMO.

The temporal evolution of both population entropy and adaptive mutation strength was monitored throughout the optimization process. As illustrated in Figure 15, the trajectory exhibits a distinct pattern, an initial transient phase characterized by rapid fluctuation, followed by a quasi-stationary regime marked by bonded, low-amplitude oscillations. This behavior provides empirical evidence that the mutation strength is dynamically modulated by information entropy. Specifically, the stabilization of mutation strength coincides with the convergence of population diversity, suggesting an effective feedback mechanism that balances exploration and exploitation as the search progresses.

**Figure 16:**
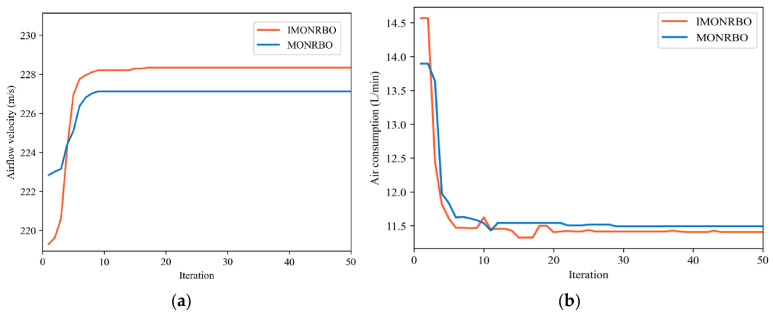
Converge curves of IMORBMO and MORBMO based on the Chebyshev KAN surrogate model.

Figure 16 depicts the iterative convergence curves for the IMORBMO and MORBMO algorithms integrated with the Chebyshev KAN surrogate model. As shown in Figure 16a, the airflow velocity converges to 228.3 m/s using IMORBMO, initiating at the eighth iteration and stabilizing by the 15th. In contrast, the MORBMO algorithm converges to 227.1 m/s, starting at the 10th iteration and stabilizing by the 20th. As shown in Figure 16b, regarding air consumption, IMRBMO achieves a minimal value of 11.1 L/min at the 25th iteration, whereas MORBMO reaches a minimum value of 11.2 L/min at the 28th iteration. Although the convergence for air consumption requires more iterations, the consistent divergence efficiency underscores the robustness of the Chebyshev KAN surrogate model. Collectively, IMORBMO demonstrates a superior capacity for global exploration across both objectives compared to MORBMO. These results validate the framework’s effectiveness in ensuring stable convergence and high-fidelity predictions.

**Figure 17:**
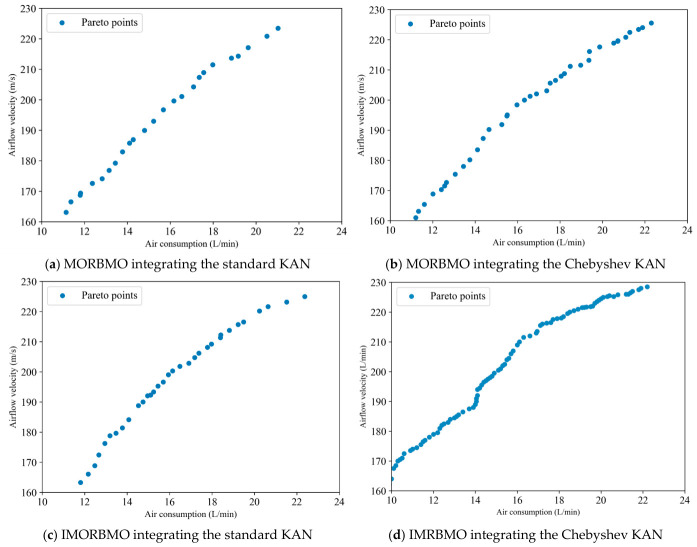
Pareto front obtained with IMORBMO or MORBMO based on the surrogate model.

To rigorously quantify the individual and synergistic contributions of the proposed IMORBMO and Chebyshev KAN framework, we conducted a comprehensive ablation study evaluating the quality of Pareto fronts across four configurations. Figure 17 illustrates the Pareto fronts obtained by the standard KAN integrated with the original MORBMO, compared against the proposed improvements. As depicted in Figure 17a, the baseline framework utilizing the standard KAN and original MORBMO, which exhibits an uneven distribution of solutions and the coverage of the Pareto front is limited to 22 L/min. In Figure 17b, the Pareto front was solved based on IMORBMO and Chebyshev KAN. Here, the integration of Chebyshev KAN rectifies the high-dimensional mapping accuracy, improving the prediction accuracy. As depicted in Figure 17c, the Pareto front generated by the standard KAN integrated with IMORBMO demonstrates that IMORBMO significantly improves population diversity, resulting in a more uniform distribution. Figure 17d presents the Pareto front obtained by the dual-improvement framework, which synergistically combines IMORBMO and Chebyshev KAN. This integrated approach merges the high-fidelity fitness landscape of Chebyshev KAN with the robust global search capability of IMORBMO, generating the most continuous and optimal Pareto front. Consequently, the trade-off curve is shifted closest to the ideal upper-left region. This comparative analysis robustly verifies that both components are indispensable and jointly contribute to the final aerodynamic optimization of the relay nozzle. In addition, the Spacing metric for MORBMO and the standard KAN surrogate model is 0.3590. MORBMO combined with Chebyshev KAN achieves a value of 0.3736, while IMORBMO combined with standard KAN yields 0.5728. IMORBMO combined with Chebyshev KAN yields 0.6087.

**Figure 18:**
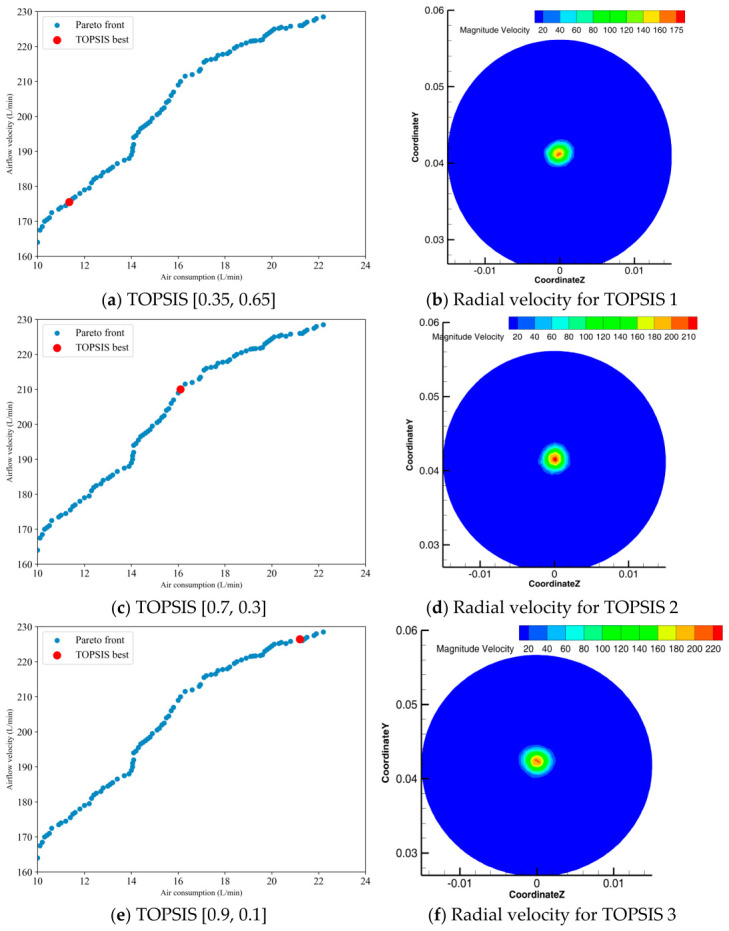
Pareto front obtained with IMORBMO based on the Chebyshev KAN surrogate model.

To quantitatively analyze the distribution characteristics and solution uniformity of the Pareto front, three representative solutions were selected based on varying TOPSIS [29] preference weights (balancing airflow velocity and air consumption) and validated via high-fidelity CFD simulations. In Figure 18a, the preference weights for airflow velocity and air consumption were set to 0.35 and 0.65, respectively. The red circle denotes the compromise solution identified by the IMORBMO algorithm on the Pareto front. According to the Pareto front and TOPSIS analysis, this optimal alternative achieves the minimum air consumption of 11.8 L/min, while the airflow velocity reaches 175.5 m/s. In Figure 18b, the maximum airflow velocity at the 15 mm cross-section area along the exit is 173.1 m/s, and air consumption is 11.5 L/min, utilizing CFD simulation. The simulation results are in excellent agreement with the prediction results obtained from the IMORBMO framework and TOPSIS decision-making technique. In Figure 18c, the weights for airflow velocity and air consumption were adjusted to 0.70 and 0.30, respectively. The corresponding optimal relay nozzle exhibits a medium air consumption of 16.1 L/min, with the airflow velocity increasing to 210.2 m/s. In Figure 18d, the maximum airflow velocity is 208.9 m/s, and air consumption is 15.8 L/min using CFD simulation. In Figure 18e, the weights for airflow velocity and air consumption were set to 0.9 and 0.1. The optimal alternative demonstrates the maximum air consumption of 21.7 L/min, while the airflow velocity achieves the maximum value of 223.1 m/s. In Figure 18f, the maximum airflow velocity at 15 mm cross-section is 220.8 m/s and air consumption is 21.5 L/min. This not only intuitively reveals the physical trade-off between airflow velocity and air consumption in relay nozzle design but also fully validates the high accuracy and decision-making flexibility of the surrogate model and developed IMORBMO multi-objective optimization framework in practical engineering applications.

**Figure 19:**
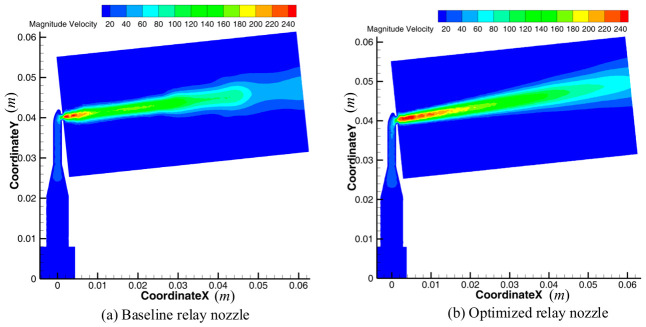
Velocity contour diagram of the original and optimized relay nozzle.

Figure 19 depicts the steady-state velocity contour on the symmetry plane for both the baseline and optimized relay nozzles. These simulations were performed using Ansys Fluent 2025.R2. The baseline relay nozzle features an outlet diameter (*d*) of 1.6 mm, an inlet diameter (D1) of 8.8 mm, a straight pipe diameter of 5.6 mm, and a spray angle of 30°. The three-dimensional fluid domain was modeled in SolidWorks 2023, followed by mesh generation and boundary condition definition in ANSYS ICEM 2025.R2. A high-quality hexahedral structured grid was generated for spatial discretization. No-slip wall boundary conditions were applied to the relay nozzle surfaces, with an inlet pressure of 0.3 MPa and outlet pressure of 101,325 Pa. Subsequently, the Reynolds-Average Navier–Stokes equations (RANS) were solved with the *k*–*ω* turbulent model under steady-state conditions, utilizing the Fluent solver. To ensure rigorous comparability between the two cases, identical mesh topologies and Fluent solver parameters were maintained. Convergences were achieved when the residuals for all governing equations fell below the prescribed thresholds. As illustrated in Figure 19, the geometric parameters of the relay nozzle have a significant impact on the steady-state flow field distribution. The jet core is clearly separated from the ambient air by a surrounding mixing layer. The jet core length of the baseline relay nozzle is approximately 10 mm, whereas that of the optimized design extends to about 20 mm, demonstrating a significant increase. This improvement of the jet core in the optimized relay nozzle can be attributed to two key geometric modifications: a reduced outlet diameter and a convergent taper angle. These changes promote stronger clustering of the jet and enhance the velocity of the jet. Consequently, the optimized geometry of the relay nozzle leads to a higher velocity than the baseline type of relay nozzle. The CFD simulation results validate the effectiveness of the proposed data-driven optimization framework in enhancing the airflow velocity of the relay nozzle.

**Figure 20:**
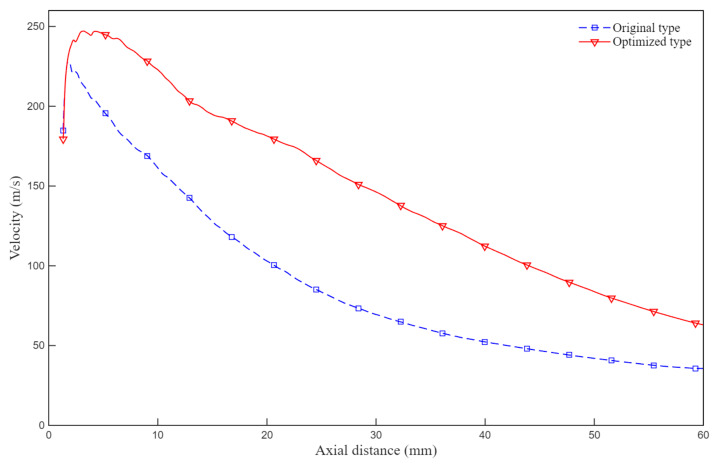
Comparison of the axial jet velocity magnitude for the original type and optimized type relay nozzle.

Figure 20 compares the axial velocity of the baseline and optimized relay nozzles obtained from CFD simulation. As shown in Figure 20, the maximum axial velocity is 217.9 m/s for the original relay nozzle, whereas the optimized design achieves a peak value of 240.0 m/s— an improvement of 10.5%. This enhancement is attributed to the optimization of geometric parameters that were obtained with the IMORBMO algorithm based on the Chebyshev KAN surrogate model. Across the axial velocity, the optimized nozzle consistently exhibits a higher axial velocity magnitude compared to the baseline. The airflow velocity accelerates to a peak value at the exit of the relay nozzle, followed by a gradual decay with increasing axial distance. Notably, the air consumption decreases from 20.2 L/min in the original type to 17.5 L/min in the optimized relay nozzle—an improvement of 15.4%. This demonstrates that the optimized version not only enhances airflow velocity but also improves energy efficiency, making the design more suitable for practical engineering.

## 6. Conclusions

This paper presents a data-driven framework that integrates a Chebyshev KAN surrogate model with an Improved Multi-objective Red-billed Blue Magpie Optimizer (IMORBMO), specifically designed to address high-dimensional, multi-objective aerodynamic optimization design problems. To accelerate the design cycle and reduce computational cost, the Chebyshev KAN surrogate model is constructed to map the geometric parameter of the relay nozzle to key performances, namely, airflow velocity and air consumption. To verify its accuracy and effectiveness, the Chebyshev KAN model is compared with conventional MLP, CNN and standard KAN models. Results demonstrate that the Chebyshev KAN surrogate model achieves significantly higher prediction accuracy while reducing the number of CFD simulations by approximately 85%. Furthermore, IMORBMO was developed utilizing an adaptive mutation mechanism and Prey-capture strategy of the Red-billed Blue Magpie algorithm, which together promote solution diversity, convergence, and ensure uniform distribution along the Pareto front. The performance of IMORBMO was rigorously evaluated on widely used ZDT and WFG benchmark suites, with comparisons of five state-of-the-art multi-objective optimizers: MOEAD, MOWOA, MODBO, MODA and MORBMO. The statistical metrics IGD, HV and GD are used to evaluate convergence, diversity, and overall solution quality. Finally, the proposed framework is applied to the aerodynamic design of a relay nozzle in an air jet loom.

The main outcomes of this article are as follows:

(1) A Chebyshev KAN surrogate model is proposed for fitting the relationship between high-dimensional structural parameters and multiple output objectives of airflow velocity and air consumption. For predicting the airflow velocity of the relay nozzle, the Chebyshev KAN achieves a coefficient of determination ($R^{2}$) of 0.975, a mean absolute error (*MAE*) of 0.103, and a root mean square error (*RMSE*) of 0.047. For predicting the air consumption of the relay nozzle, the Chebyshev KAN yields an exceptional predictive performance, with a coefficient of determination ($R^{2}$) of 0.950, a mean absolute error (*MAE*) of 0.115, and a root mean square error (*RMSE*) of 0.056
(2) For ZDT and WFG problems, IMORBMO demonstrates strong performance, with the lowest IGD and GD metrics and the highest HV metrics. Compared with the state-of-the-art algorithms, IMORBMO exhibits superior convergence, diversity, and uniformity in the convex, non-convex and nonlinear discontinuous Pareto fronts. These results highlight IMORBMO is effective, stable, and reliable in addressing complex trade-offs among multiple conflicting objectives.
(3) The integration of Chebyshev KAN and IMORBMO creates a robust framework for aerodynamic design of the relay nozzle. The optimal aerodynamic performance was achieved at a maximum airflow velocity of 240.0 m/s and air consumption of 17.5 L/min. The corresponding optimal structural parameters are as follows: input diameter (D1) of 7.6 mm, the straight pipe diameter of 5.8 mm, the outlet diameter of 1.7 mm and spray angle of 40°.

Building on the findings, the proposed data-driven optimization framework is excellent in solving multi-objective optimization problems like aerodynamic design in textile components. Further investigation is warranted to assess the applicability of the proposed framework to more complex scenarios, including higher-dimensional input features and more complex engineering designs incorporating nonlinear constraints.

## Figures and Tables

**Table 1:** Relay nozzle structure parameters and air consumption.

Number	*d* (mm)	*D*1 (mm)	*D*2 (mm)	*α* (°)	*V* (m/min)	*Q* (L/min)
1	0.8	9.0	7.2	60	94.4	3.21
2	0.8	9.0	8.4	60	92.9	3.63
3	0.8	10.0	6.8	60	97.2	4.08
4	1.2	10.0	6.8	60	151.7	10.36
5	1.2	10.8	5.8	32	157.4	13.58
6	1.6	9.6	4.8	16	200.6	15.87
7	1.6	10.6	8.0	16	187.8	20.75
8	1.6	8.8	6.4	16	220.6	21.28
9	1.8	9.2	7.2	60	232.8	24.52
10	1.8	12.0	10.0	32	193.2	27.38

**Table 2:** Evaluation metrics for various surrogate models on test data.

		Evaluation Metric			Calculation Time	
MODEL		Relative Mean Error (*MAE*)	Root Mean Square Error (*RMSE*)	Determination Coefficient (*R*^2^)	Training Time (s)	Inference Latency (ms/Sample)
MLP	*V* (m/s)	10.221	7.945	0.882	24.970	2.538
	*Q* (L/min)	3.502	2.531	0.720		
CNN	*V* (m/s)	8. 856	7.128	0.874	20.294	3.013
	*Q* (L/min)	3.348	2.046	0.738		
Kriging	*V* (m/s)	2.789	3.414	0.901	14.852	2.081
	*Q* (L/min)	1.013	1.268	0.929		
SVR	*V* (m/s)	1.268	1.916	0.884	13.512	2.450
	*Q* (L/min)	6.68	5.309	0.916		
RBF	*V* (m/s)	1.018	1.359	0.942	10.461	2.211
	*Q* (L/min)	2.971	1.575	0.881		
KAN	*V* (m/s)	8.431	6.497	0.883	11.565	2.083
	*Q* (L/min)	2.111	1.303	0.907		
Chebyshev KAN	*V* (m/s)	0.103	0.047	0.975	9.495	1.369
	*Q* (L/min)	0. 115	0.056	0.950		

**Table 3:** The IGD metrics of all compare algorithms on ZDT and WFG functions.

		MOEAD	MOWOA	MODBO	MODA	MORBMO	IMORBMO
ZDT1	*f_mean_*	1.660 × 10^−2^	3.016 × 10^−3^	4.884 × 10^−1^	4.525 × 10^−2^	3.180 × 10^−2^	2.746 × 10^−3^
	*f_std_*	6.547 × 10^−2^	1.946 × 10^−3^	1.628 × 10^−3^	1.119 × 10^−2^	3.426 × 10^−1^	1.581 × 10^−3^
ZDT2	*f_mean_*	8.809 × 10^−1^	4.874 × 10^−2^	4.218 × 10^−1^	5.782 × 10^−1^	4.266 × 10^−1^	3.452 × 10^−2^
	*f_std_*	2.685 × 10^−1^	3.383 × 10^−4^	4.121 × 10^−3^	1.442 × 10^−2^	4.800 × 10^−4^	1.621 × 10^−4^
ZDT3	*f_mean_*	3.819 × 10^−1^	5.618 × 10^−1^	6.021 × 10^−1^	4.902 × 10^−1^	5.993 × 10^−1^	3.393 × 10^−1^
	*f_std_*	4.057 × 10^−2^	2.951 × 10^−3^	4.311 × 10^−2^	2.661 × 10^−2^	1.080 × 10^−3^	7.242 × 10^−4^
WFG2	*f_mean_*	2.963 × 10^−1^	2.851 × 10^−1^	4.815 × 10^−1^	2.288 × 10^−1^	1.444 × 10^−1^	1.113 × 10^−1^
	*f_std_*	2.395 × 10^−2^	3.151 × 10^−2^	2.245 × 10^−2^	2.567 × 10^−2^	6.610 × 10^−2^	2.083 × 10^−2^
WFG3	*f_mean_*	4.619 × 10^−2^	5.594 × 10^−2^	3.816 × 10^−2^	2.040 × 10^−1^	2.946 × 10^−2^	6.624 × 10^−3^
	*f_std_*	1.169 × 10^−2^	4.556 × 10^−3^	2.002 × 10^−3^	2.356 × 10^−2^	1.560 × 10^−3^	2.644 × 10^−4^
WFG4	*f_mean_*	7.856 × 10^−2^	7.635 × 10^−2^	7.049 × 10^−2^	1.034 × 10^−1^	2.303 × 10^−2^	2.094 × 10^−2^
	*f_std_*	3.571 × 10^−3^	3.673 × 10^−3^	2.464 × 10^−4^	1.634 × 10^−2^	1.284 × 10^−3^	1.574 × 10^−4^

**Table 4:** The HV metrics of all compare algorithms, ZDT, and WFG functions.

		MOEAD	MOWOA	MODBO	MODA	MORBMO	IMORBMO
ZDT1	*f_mean_*	5.150 × 10^−1^	7.203 × 10^−1^	7.189 × 10^−1^	4.525 × 10^−1^	7.180 × 10^−1^	8.746 × 10^−1^
	*f_std_*	7.554 × 10^−2^	2.840 × 10^−2^	1.771 × 10^−2^	1.119 × 10^−2^	3.426 × 10^−1^	1.581 × 10^−3^
ZDT2	*f_mean_*	2.624 × 10^−1^	4.032 × 10^−1^	4.118 × 10^−1^	2.782 × 10^−1^	4.266 × 10^−1^	5.374 × 10^−1^
	*f_std_*	2.882 × 10^−2^	8.148 × 10^−2^	4.121 × 10^−2^	1.842 × 10^−2^	4.800 × 10^−3^	1.221 × 10^−3^
ZDT3	*f_mean_*	4.040 × 10^−1^	5.986 × 10^−1^	6.001 × 10^−1^	4.302 × 10^−1^	5.893 × 10^−1^	1.328 × 10^0^
	*f_std_*	6.609 × 10^−2^	6.063 × 10^−4^	4.311 × 10^−3^	2.661 × 10^−2^	3.080 × 10^−4^	2.242 × 10^−4^
WFG2	*f_mean_*	5.609 × 10^−1^	1.256 × 10^−1^	6.252 × 10^−1^	5.311 × 10^−1^	6.140 × 10^−1^	6.132 × 10^0^
	*f_std_*	3.359 × 10^−2^	2.563 × 10^−2^	2.268 × 10^−3^	1.947 × 10^−2^	1.385 × 10^−3^	1.161 × 10^−3^
WFG3	*f_mean_*	5.617 × 10^−1^	5.023 × 10^−1^	5.641 × 10^−1^	4.590 × 10^−1^	5.577 × 10^−1^	5.638 × 10^0^
	*f_std_*	7.319 × 10^−3^	3.211 × 10^−3^	1.596 × 10^−3^	1.018 × 10^−2^	5.824 × 10^−3^	1.482 × 10^−4^
WFG4	*f_mean_*	3.107 × 10^−1^	3.050 × 10^−1^	3.072 × 10^−1^	2.909 × 10^−1^	3.011 × 10^−1^	3.346 × 10^0^
	*f_std_*	3.602 × 10^−3^	3.402 × 10^−3^	9.644 × 10^−3^	8.224 × 10^−2^	9.741 × 10^−3^	2.285 × 10^−3^

**Table 5:** The GD metrics of all compare algorithms, ZDT, and WFG functions.

		MOEAD	MOWOA	MODBO	MODA	MORBMO	IMORBMO
ZDT1	*f_mean_*	1.187 × 10^−2^	5.921 × 10^−3^	6.892 × 10^−3^	7.485 × 10^−2^	6.150 × 10^−3^	4.091 × 10^−3^
	*f_std_*	4.689 × 10^−3^	2.449 × 10^−3^	2.256 × 10^−3^	3.425 × 10^−2^	3.001 × 10^−3^	2.081 × 10^−4^
ZDT2	*f_mean_*	5.693 × 10^−2^	5.449 × 10^−2^	4.567 × 10^−3^	1.542 × 10^−1^	6.825 × 10^−3^	4.161 × 10^−3^
	*f_std_*	1.657 × 10^−2^	6.842 × 10^−3^	2.653 × 10^−4^	6.421 × 10^−2^	4.401 × 10^−5^	3.837 × 10^−5^
ZDT3	*f_mean_*	2.161 × 10^−1^	2.596 × 10^−3^	4.653 × 10^−3^	6.611 × 10^−2^	1.910 × 10^−3^	1.561 × 10^−3^
	*f_std_*	3.567 × 10^−3^	4.988 × 10^−3^	3.345 × 10^−2^	2.521 × 10^−2^	1.222 × 10^−2^	5.451 × 10^−4^
WFG2	*f_mean_*	5.433 × 10^−2^	7.365 × 10^−2^	1.367 × 10^−1^	2.681 × 10^−1^	2.410 × 10^−3^	1.624 × 10^−3^
	*f_std_*	5.067 × 10^−3^	1.834 × 10^−3^	2.458 × 10^−3^	1.260 × 10^−2^	7.403 × 10^−4^	5.292 × 10^−4^
WFG3	*f_mean_*	1.165 × 10^−3^	6.177 × 10^−3^	3.895 × 10^−3^	2.111 × 10^−2^	8.260 × 10^−4^	1.321 × 10^−4^
	*f_std_*	8.978 × 10^−3^	9.319 × 10^−4^	2.559 × 10^−4^	5.001 × 10^−3^	1.480 × 10^−4^	1.822 × 10^−5^
WFG4	*f_mean_*	4.311 × 10^−2^	6.990 × 10^−3^	6.417 × 10^−2^	1.010 × 10^−2^	6.493 × 10^−3^	5.225 × 10^−3^
	*f_std_*	7.993 × 10^−3^	6.790 × 10^−4^	5.269 × 10^−3^	1.251 × 10^−3^	9.200 × 10^−4^	4.951 × 10^−4^

## Data Availability

The source code of IMORBMO and data are available at https://github.com/Wuse-MM/mo (accessed on 1 April 2026).

## Abbreviations

The following abbreviations are used in this manuscript:

CFD	Computational Fluid Dynamics
Chebyshev KAN	Chebyshev Kolmogorov–Arnold Network
CNN	Convolutional Neural Network
GD	Generational Distance
HV	Hypervolume
IGD	Inverted Generational Distance
IMORBMO	Improved Multi-objective Red-billed Blue Magpie Optimizer
KAN	Kolmogorov–Arnold Network
MAE	Mean Absolute Error
MLP	Multilayer Perceptron
MO	Multi-Objective
MODA	Multi-Objective Dragonfly Algorithm
MODBO	Multi Objective Dung Beetle Optimizer
MOEAD	Multi-objective Evolutionary Algorithm based on Decomposition
MORBMO	Multi-objective Red-billed Blue Magpie Optimizer
MOWOA	Multi-objective Whale Optimization Algorithm
PF	Pareto front
RANS	Reynolds-Average Navier–Stokes equations
RMSE	Root Mean Square Error
WFG	Walking Fish Group test suite
ZDT	Zitzler-Deb-Thiele test suite
